# The value of ^18^F-NaF PET/CT in the diagnosis of bone metastases in patients with nasopharyngeal carcinoma using visual and quantitative analyses

**DOI:** 10.3389/fbioe.2022.949480

**Published:** 2022-08-24

**Authors:** Dong Wang, HaiWen Li, ChengMao Guo, Shisang Huang, XuFeng Guo, JingXing Xiao

**Affiliations:** ^1^ Department of Nuclear Medicine (PET-CT center), Affiliated Hospital of Guangdong Medical University, ZhangJiang, China; ^2^ Cancer Center, Affiliated Hospital of Guangdong Medical University, ZhangJiang, China

**Keywords:** PET/CT, 18F-sodium fluoride, nasopharyngeal carcinoma, bone metastases, visual assessment, quantitative analysis, standardized uptake value

## Abstract

To assess the diagnostic value of ^18^F-NaF PET/CT in diagnosing bone metastases in patients with nasopharyngeal carcinoma (NPC) using visual and quantitative analyses. 164 patients with NPC who underwent ^18^F-NaF PET/CT between 2017 and 2021 were included. The sensitivity, specificity, and accuracy were calculated. All bone lesions were divided into metastatic bone lesion group and benign lesion group; the benign lesion group was further subdivided into benign lesion without osteophyte and fracture group (CT images showing no osteophyte, no fracture), and benign lesion with osteophyte and fracture group (CT images showing typical osteophytes and fractures), the differences in maximum standardized uptake value (SUVmax) were compared between every two groups, and the diagnostic cut-off values were derived from receiver operating characteristic curves (ROC). Quantitative data were expressed as mean ± SD, multiple continuous variables were compared using one-way analysis of variance (ANOVA), and multiple comparisons among more than two groups were made using the Bonferroni method. The sensitivity, specificity, and overall accuracy of ^18^F-NaF PET/CT for the diagnosis of bone metastases in NPC were 97.1%, 94.6%, and 95.1% based on the patient level and 99.5%, 91.5%, and 96.4% based on the lesion level, respectively. The SUVmax was significantly different between the metastatic bone lesion group and the benign lesion without osteophyte and fracture group (*p* < 0.05); the area under the curve was 0.865, the threshold was 12.5, the sensitivity was 0.912, and the specificity was 0.656. Visual analysis of ^18^F-NaF PET/CT has high sensitivity and specificity for diagnosing bone metastases in NPC. After excluding osteophytes and fracture lesions based on CT findings, using SUVmax ≥12.5 as the threshold can be an important reference for the differential diagnosis of bone metastases and benign bone lesions in patients with NPC.

## Introduction

The incidence of nasopharyngeal carcinoma (NPC) geographically varies, and its highest incidence was reported in southern China. The risk of distant metastasis is even higher in patients with T4 and N3 stages, and the rate of distant metastasis is as high as 45–60% in patients who died. The more frequent sites of distant metastasis were bones, lungs, and non-cervical lymph nodes (Shen L. J et al., 2015).

Several patients with metastatic bone tumors are associated with bone-related adverse events, such as pathological fractures, pain, spinal cord compression, spinal instability, and hypercalcemia (Xu et al., 2016; Yang et al., 2019). Once NPC develops to bone metastases, the clinical stage is elevated from M0 to M1, and this change may significantly influence the adjustment of treatment strategies and patient prognosis. Consequently, the originally planned radiotherapy-based treatment strategy may be adjusted to the combination of chemotherapy-based treatment strategies (Chen et al., 2019). Therefore, early detection of patients with bone-metastatic NPC and determination of the site and number of bone metastases is crucial.

As a molecular imaging technique, positron emission tomography/computed tomography (PET/CT) can obtain metabolic information about malignant tumors (Ayati et al., 2021; Han and Choi, 2021). The ^18^F-NaF has been used for 40 years as an oleophilic contrast agent and is a positron imaging agent mainly for bone imaging, and its application was somewhat limited before the advent of PET/CT. Besides, the ^18^F-NaF has better pharmacokinetic properties than the contrast agent ^99m^Tc-MDP for bone imaging, and with the development of PET/CT, ^18^F-NaF has gradually attracted radiologists’ attention (Grant et al., 2008; Segall et al., 2010). In 2015, the European Society of Nuclear Medicine issued guidelines for the clinical application of ^18^F-NaF PET/CT for bone imaging successively (Beheshti et al., 2015).^18^F-NaF PET/CT imaging of various malignant tumors, such as prostate cancer (Langsteger et al., 2016), breast cancer (Arvola et al., 2019), and lung cancer (Rao et al., 2016) has been conducted, which showed its potential application value. We have previously investigated the Observer agreement and accuracy of ^18^F-NaF PET/CT in the diagnosis of bone metastases in NPC (Xiao et al., 2020). However, only a few studies with a small sample size have concentrated on ^18^F-NaF PET/CT imaging of NPC (Zhang et al., 2018), and no quantitative study has been carried out. Thus, the present study aimed to investigate the diagnostic value of ^18^F-NaF PET/CT in the diagnosis of bone metastases in patients with NPC using visual and quantitative analyses in large samples. We hypothesized that ^18^F-NaF PET/CT has high diagnostic efficacy in diagnosing bone metastases and that SUVmax is valuable in differentiating bone metastases from benign bone lesions in NPC.

## Materials and methods

### Patients

**FIGURE 1:**
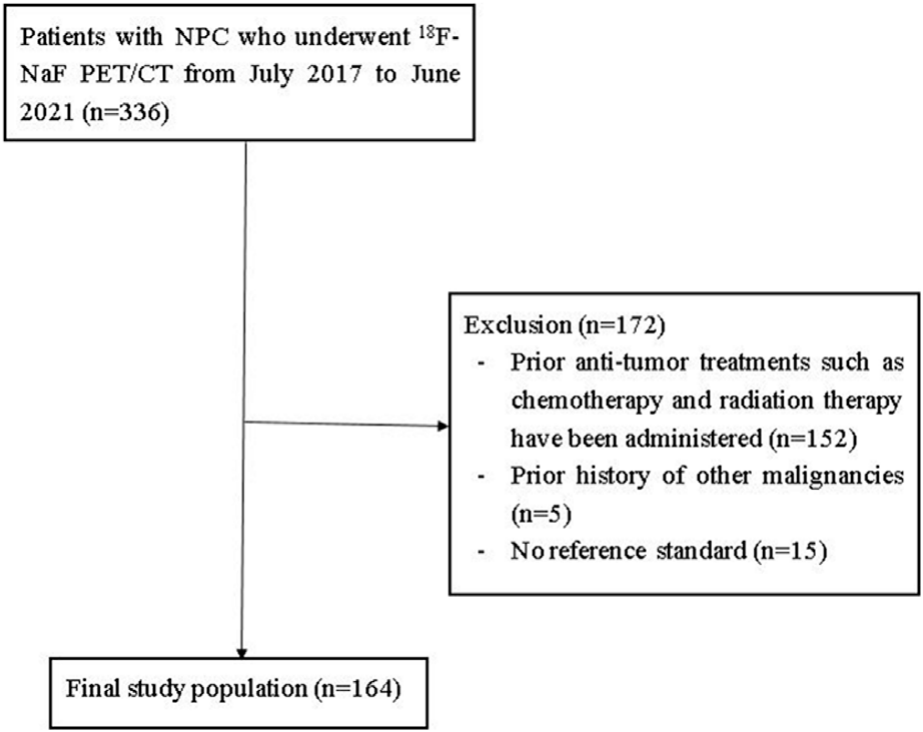
Flow diagram of patient selection and exclusion criteria for the initial study.

We retrospectively reviewed the images of NPC patients who underwent ^18^F-NaF PET/CT in our center from 1 July 2017 and 31 June 2021. The inclusion criteria were as follows: i. Patients who were histopathology confirmed with NPC; ii. Performing whole-body ^18^F-NaF PET/CT before NPC therapy; iii. Availability of standardized treatment and clinical and imaging follow-up data for more than half a year, including CT, magnetic resonance imaging (MRI), and PET/CT findings; iv. Patients without other serious complications or other types of cancer. A total of 164 patients were enrolled. The study included 124 men and 40 women aged 21 to 71 (average age, 51.2) years old. Patients were staged according to the 8th edition of the American Joint Committee on Cancer (AJCC) TNM staging system. This retrospective study was approved by the Ethics Committee of Affiliated Hospital of Guangdong Medical University. A flow diagram summarizing the initial candidates and each exclusion procedure are shown in Figure 1.

### ^18^F-NaF PET/CT

^18^F-NaF PET/CT was conducted in accordance with the guidelines from the Nuclear Medicine and the European Association of Nuclear Medicine. Patients received an intravenous injection of approximately 200 MBq of ^18^F-NaF. The PET data were acquired at 64±6 (mean ± standard deviation (SD) min after the tracer injection using the GE Discovery Elite 690 PET/CT scanner (General Electric Medical Systems, Milwaukee, WI, United States). Low-dose helical CT transmission scan was carried out (pitch, 0.984, current, 120–230 mAs; voltage, 120 kV; display field of view (DFOV), 50.0 cm; slice thickness, 3.75 mm; reconstruction slice, 1.25 mm). The static emission scan was performed over the whole body with 2-min (the GE Discovery-690 PET/CT scanner) acquisition times per bed position. The sinogram data of CT were corrected for dead time, decay, and photon attenuation and reconstructed in a 128×128 matrix. Image reconstruction followed a fully three-dimensional (3D) maximum likelihood ordered subset expectation-maximization algorithm.

### Definition of bone metastases and benign bone lesions

**Bone Metastases**: the areas of focally increased ^18^F-NaF uptake were recorded as malignant unless a benign etiology (e.g., degenerative changes or hemangioma) for this uptake was identified at the same location on the corresponding CT images. The CT component of PET/CT was used to determine whether bone lesions identified on PET had an osteoblastic or osteolytic appearance. Bone destruction or osteoblastic manifestation of bone (local and asymmetric lesions with increased density) with NaF uptake was targeted as malignancy. Bone metastases are classified into four types: osteolytic, osteogenic, mixed and without significant changes based on CT images.

**Benign bone lesions:** localized abnormally concentrated radioactive lesions with a history of trauma, surgery, radiation therapy, etc., and typical fractures, osteophytes and benign bone tumors on CT defined as benign bone lesions. Osteomalacia/degeneration was defined as intervertebral disc degeneration in the form of vacuum sign, labral hyperplasia and sclerosis at the margins of the vertebral body; hyperplasia and sclerosis of the intervertebral small joint and sacroiliac joint surfaces. Fracture is defined as disruption of cortical continuity, or multiple fragmented fracture fragments, displacement of the fracture may also be seen, and bone repair/scab formation after follow-up.

### Reference standard

The reference standard followed Jadvar et al.‘s research method (Jadvar et al., 2012). The final bone metastasis of a given site was determined based on either pathological examination from CT-guided or surgical biopsies or the results of follow-up by MRI, contrast-enhanced CT or PET/CT for more than 6 months for every patient. The suspicious lesions detected by PET/CT were confirmed to be metastasis when the tissues were pathologically proved to be metastatic, or the lesions became larger during the follow-up periods or decreased in size after treatment. On the contrary, they were diagnosed as non-metastatic lesions when no change in size was observed during follow-up examinations. The final diagnosis was arrived at by consensus at a conference held by the multidisciplinary group of NPC in our hospital.

### Statistical analysis

All data were analyzed based on two levels, including the lesion level and the patient level. Indicators of diagnostic performance were expressed using dichotomous variables. Diagnostic performance was evaluated using positive-predictive value (PPV), negative-predictive value (NPV), sensitivity, specificity, and accuracy.

Quantitative data were expressed as mean ± SD; multiple continuous variables were compared using one-way analysis of variance (ANOVA), and multiple comparisons among more than two groups were made using the Bonferroni method; a standardized uptake value (SUV) threshold was proposed to be used as a cut-off for diagnosing metastatic bone lesions versus benign bone lesions (non-osteophytes, non-fractures, etc.). Using receiver operating characteristic (ROC) curves. The statistical analysis was performed using R (version. 3.6.3; www.r-project.org) software, and *p* < 0.05 was considered statistically significant.

## Results

### Patients’ clinical characteristics

A total of 164 patients were included in the analysis. Thirty-five (21.3%) patients with NPC developed bone metastases, with 444 metastatic bone lesions and 281 benign bone lesions. The most common sites of metastatic osseous lesions were ribs (n = 94), followed by pelvic bones (*n* = 93). Patients’ clinical characteristics are summarized in Table 1.

**TABLE 1:** Basic data characteristics of 164 cases of nasopharyngeal carcinoma.

Characteristics	(*n* = 164)
Gender	
Male	124
Female	40
Age (Mean ± standard deviation)	50.132±11.95
Pathological typing	
Undifferentiated non-keratinizing carcinoma	160
Differentiated non-keratinizing carcinoma	4
M-staging	
M0	128
M1	36
TNM staging	
Ⅰ	0
Ⅱ	14
Ⅲ	70
Ⅳa	44
Ⅳb	36
Bone metastasis area (site)	444
Ribs	94
Pelvic bone	93
Cervical Spine	30
Thoracic spine	85
Lumbar spine	55
Scapula	32
Limb bone	26
Skull	13
Otheres	16

### Visual evaluation of ^18^F-NaF PET/CT images

Each patient’s ^18^F-NaF PET/CT images were independently interpreted by two board-certified nuclear medicine physicians. Both physicians (with experience in interpreting more than 1000 ^18^F-NaF PET/CT images) were blinded to the objectives of the study. If there was a discrepancy between the two readers, the opinion of a third expert with more than 10 years of nuclear medicine certification was included, and ultimately to judge the nature of the lesion by the vote of the three experts. The diagnostic efficacy of ^18^F-NaF PET/CT imaging of bone metastases in patients with NPC was assessed using visual analysis. The visual analysis revealed that sensitivity, specificity, and accuracy of ^18^F-NaF PET/CT imaging were 97.1%, 94.6%, and 95.1% based on the patient level and 99.5%, 91.5%, and 96.4% based on the lesion level, respectively (Table 2).

**TABLE 2:** The diagnostic results of visual analysis for diagnosing bone metastases (n = 164).

	Sensitivity (95%CI)	Specificity (95%CI)	Accuracy (95%CI)	PPV(95%CI)	NPV(95%CI)
Patient-based Level	0.971 (0.914, 1.000)	0.946 (0.907, 0.977)	0.951 (0.915, 0.982)	0.829 (0.733, 0.921)	0.992 (0.975, 1.000)
Lesion-based Level	0.995 (0.989, 1.000)	0.915 (0.883, 0.943)	0.964 (0.950, 0.977)	0.948 (0.930, 0.967)	0.992 (0.981, 1.000)

PPV, positive predictive value; NPV, Negative predictive value.

### Quantitative evaluation of ^18^F-NaF PET/CT imaging

**FIGURE 2:**
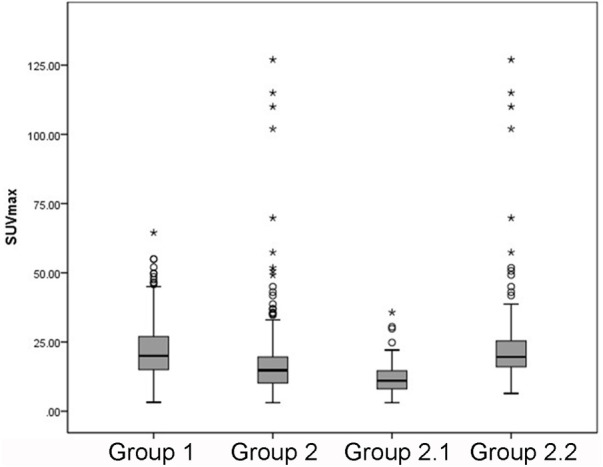
The distribution of SUVmax of bone lesions.

A total of 164 patients with 725 bone lesions were divided into metastatic bone lesion group (n = 444, defined as group 1) and benign lesion group (n = 281, defined as group 2); benign lesion group was further subdivided into benign lesion without osteophyte and fracture group (CT images showing no osteophyte, no fracture, defined as group 2.1), and benign lesion with osteophyte and fracture group (CT images showing typical osteophytes and fractures, e.g. the lesion is around the vertebral body, or a distinct fracture line is visible, defined as group 2.2), and the number of bone lesions in each group is presented in Table 3. Table 4 shows the results of a two-by-two comparison of SUVmax in each group. The results showed that there was no statistically significant difference between groups 1 and 2.2 (group 1 vs group 2.2, *p* = 0.268); between each pair of the remaining groups, significant differences were found [(group 1 vs group 2, *p* < 0.001) (group 1 vs group 2.1, *p* < 0.001) (group 2 vs group 2.1, *p* < 0.001) (group 2 vs group 2.2, *p* < 0.001) (group 2.1 vs groups 2.2, *p* < 0.001)]. The box plots of the distribution of SUVmax of bone lesions in the four groups are shown in Figure 2.

**TABLE 3:** SUVmax for each group of bone lesions.

Group	Number	Mean	Std. Deviation	Std. Error	95% CI		Minimum	Maximum
					Lower Bound	Upper Bound		
1	444	22.2286	9.42603	0.44734	21.3494	23.1078	3.2	64.5
2	281	17.5573	14.88028	0.88768	15.8099	19.3047	3.1	127
2.1	154	11.6539	5.37034	0.43275	10.799	12.5088	3.1	35.7
2.2	127	24.7157	19.04692	1.69014	21.371	28.0605	6.4	127

**TABLE 4:** Comparison of SUVmax between groups of bone lesions.

Group	Group	Mean Difference	Std. Error	*p* Value	95% CI	
					Lower Bound	Upper Bound
1	2	4.67131*	0.93729	0	2.1936	7.149
	2.1	10.57471*	1.14986	0	7.535	13.6144
	2.2	−2.48714	1.23729	0.268	−5.7579	0.7836
2	1	−4.67131*	0.93729	0	−7.149	−2.1936
	2.1	5.90340*	1.23276	0	2.6446	9.1622
	2.2	−7.15845*	1.31469	0	−10.6338	−3.6831
2.1	1	−10.57471*	1.14986	0	−13.6144	−7.535
	2	−5.90340*	1.23276	0	−9.1622	−2.6446
	2.2	−13.06185*	1.4738	0	−16.9579	−9.1659
2.2	1	2.48714	1.23729	0.268	−0.7836	5.7579
	2	7.15845*	1.31469	0	3.6831	10.6338
	2.1	13.06185*	1.4738	0	9.1659	16.9579

* The mean difference is significant at the 0.05 level. CI, Confidence Interval.

**FIGURE 3:**
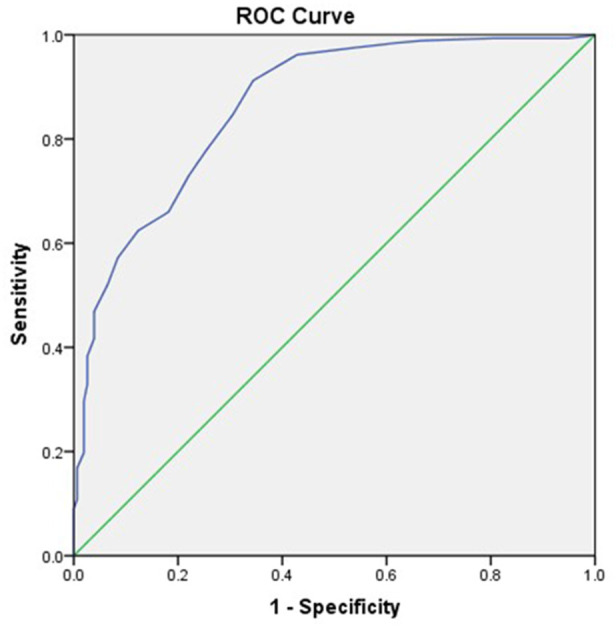
The ROC curves of groups 1 and 2.1.

The ROC curves of group 1 and group 2.1 are illustrated in Figure 3. It was revealed that the values of area under the curve (AUC), sensitivity, specificity, and the diagnostic cut-off value of SUVmax were 0.865 (95% CI: 0.831–0.899), 0.912, 0.656, and 12.5 g/ml, respectively (Table 5).

**TABLE 5:** The characteristics of ROC curves.

Characteristic	Value
Cutoff point	12.500
Sensitivity	0.912
Specificity	0.656
AUC	0.865
95% CI of AUC	(0.831, 0.899)

The metastatic bone lesions were differentiated according to their size, including 277 lesions with a diameter of ≥2 cm and 167 lesions with a diameter of <2 cm. The mean values of SUVmax of lesions in the two groups were 25.77±9.41 g/ml (≥2 cm group), and 16.35±5.75 g/ml (<2 cm group), respectively; the SUVmax of the lesion diameter ≥2 cm group was significantly higher than that of the lesion diameter <2 cm group (t = -11.66, *p* < 0.01). The metastatic bone lesions were differentiated according to their pattern according to CT findings, including 231 osteolytic lesions, 91 osteogenic lesions, and 122 lesions with no obvious changes on CT. The mean values of SUVmax of lesions in the three groups were 17.03±5.22 g/ml (osteolytic lesions), 29.27±10.12 g/ml (osteogenic lesions), and 26.82 ± 8.10 g/ml (no obvious changes in CT), respectively; the SUVmax of the osteogenic lesions group and lesions with no obvious changes on CT group were significantly higher than that of the osteolytic lesions group (*p* < 0.01).

## Discussion

Based on visual analysis, this study demonstrated that ^18^F-NaF PET/CT has excellent diagnostic accuracy in diagnosing bone metastases in patients with NPC. 35 (21.3%) patients with NPC developed bone metastases, the most common site of bone metastasis was the rib cage, followed by the pelvis. In addition, this study revealed that quantitative NaF PET/CT is valuable in diagnosing bone metastases from NPC. To the best of our knowledge, this study was the first to perform a quantitative analysis of ^18^F-NaF PET/CT findings for the diagnosis of bone metastases in patients with NPC.

In this study, the sensitivities were 97.1% and 99.5% at the patient level and at the lesion level using visual analysis, respectively. A Meta-analysis (Shen C. T et al., 2015) demonstrated that the sensitivity of ^18^F-NaF PET/CT for the diagnosis of bone metastases in various cancer was 92% at the patient level and 87% at the lesion level. Zhang et al. (Zhang et al., 2018) found that the sensitivity of ^18^F-NaF PET/CT for detection of bone metastasis in 45 NPC patients was 98.3%, which was in line with our findings. The reasons for this high sensitivity are as follows. Firstly, ^18^F-NaF is an excellent radiopharmaceutical for bone imaging because fluoride ions are an analogue of the hydroxyl group found in the hydroxyapatite bone crystals, which exchange with hydroxyl groups in hydroxyapatite bone crystals to form fluorapatite (Grant et al., 2008). Secondly, it detects the presence of lesions directly by bone mineral metabolism rather than indirectly showing lesion involvement owing to the increased bone mineral turnover, especially in lesions with pathologic changes (Even-Sapir et al., 2004; Iagaru et al., 2013).

**FIGURE 4:**
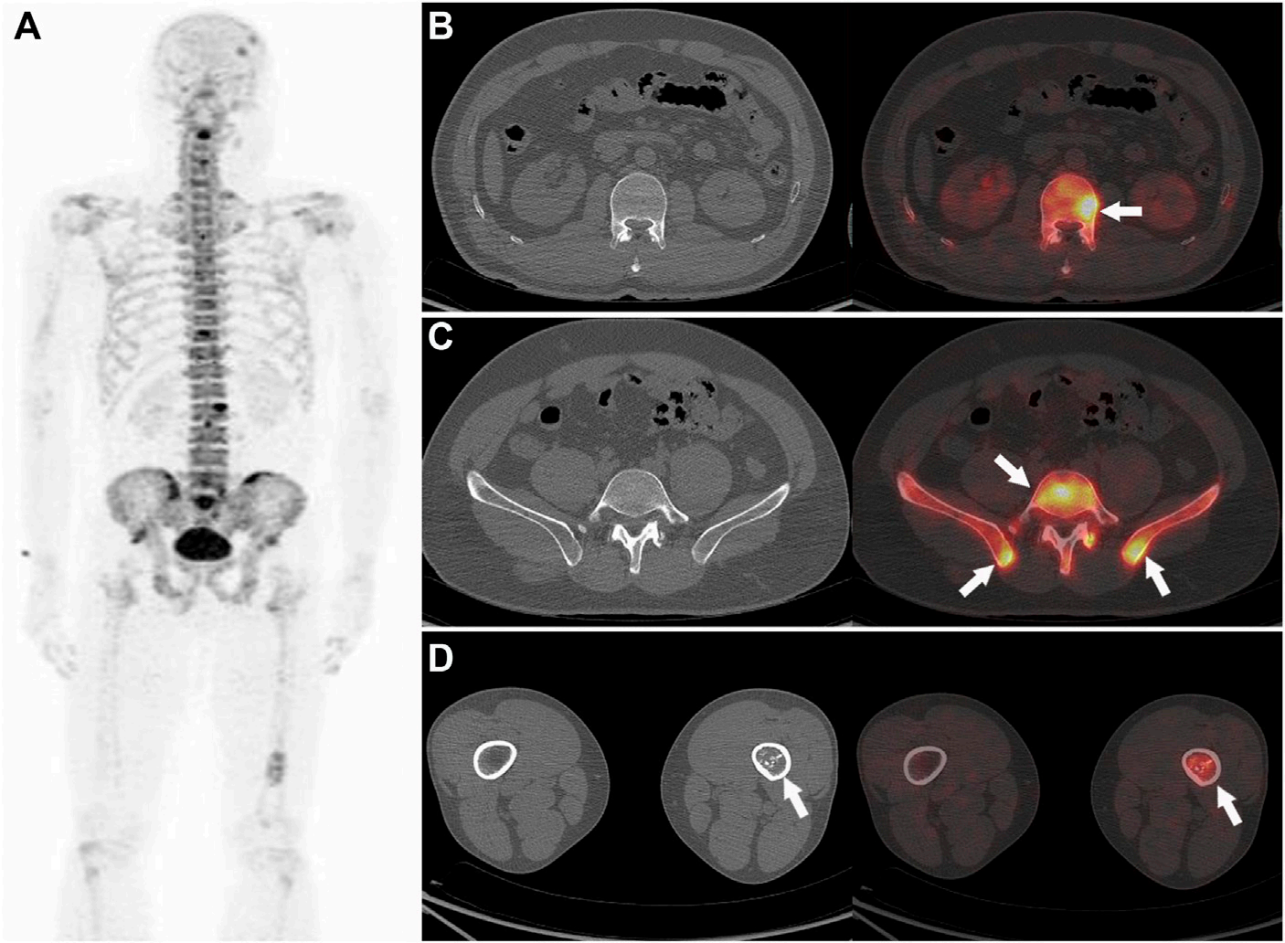
Nasopharyngeal carcinoma (T3M3M1) in a 42-year-old male patient. The PET MIP **(A)** shows multiple ^18^F-NaF uptakes in the skull, spine, pelvis, and the left tibia. Multiple abnormal ^18^F-NaF uptakes in the spine **(B)**, arrow and pelvis **(C)**, arrow, no abnormal morphology changes can be observed in the corresponding CT, were diagnosed as bone metastases (correctly according to the reference standard). A mild abnormal ^18^F-NaF uptake in the left tibia (D, arrow), and a heterogeneous increase in density in the corresponding CT **(D)**, which were diagnosed as abnormal bone fibre proliferation disease (correctly according to the reference standard).

In addition to high sensitivity, our study also showed a noticeable specificity of ^18^F-NaF PET/CT, which was 94.6% at the patient level and 91.5% at the lesion level, and was close to the results of previous studies (Shen C. T et al., 2015; Zhang et al., 2018; Liu et al., 2019), mainly relying on the exclusion of benign lesions (e.g. fractures and osteophytes, from CT images). PET has been proved to be an effective tool in the management of malignant tumor patients, and it provides limited information on bone lesion morphologic abnormalities. Differentiation between benign lesions and malignant bone lesions is obtained by further CT validation (Figure 4). The remarkable nuclear medicine technological developments in positron imaging devices combined with coregistration CT have resulted in a renewed interest in^18^F-NaF. Thus, ^18^F-NaF PET/CT detection can provide precise information regarding both the morphologic and bone mineral metabolism changes occurring in bone metastases, so the specificity of ^18^F-NaF PET in bone metastases detection can be improved by the use of the PET-CT system (Hawkins et al., 1992; Even-Sapir et al., 2004; Grant et al., 2008; Rao et al., 2016). However, as ^18^F-NaF is a non-bone metastasis-specific imaging agent, there were a few misdiagnoses that occurred in our study, including seven patients with false-positive test results with a false-positive rate of 5.4% (7/129) and 26 false-positive lesions with a false-positive rate of 9.25%. The reason for these results may be that some benign bone lesions do not have a high degree of NaF tracer uptake (only mildly increased uptake), and CT images do not show typical features of benign lesions (e.g., no osteophyte, no fracture, or degenerative changes), and there is an overlap between such lesions and bone metastases in terms of CT findings and PET of bone metabolism. Hence, Nuclear medicine physicians need to be cautious in drawing conclusions about metastatic bone lesions when a bone lesion is atypical in ^18^F-NaF PET/CT finding, in which further examination or a longer follow-up may be required.

**FIGURE 5:**
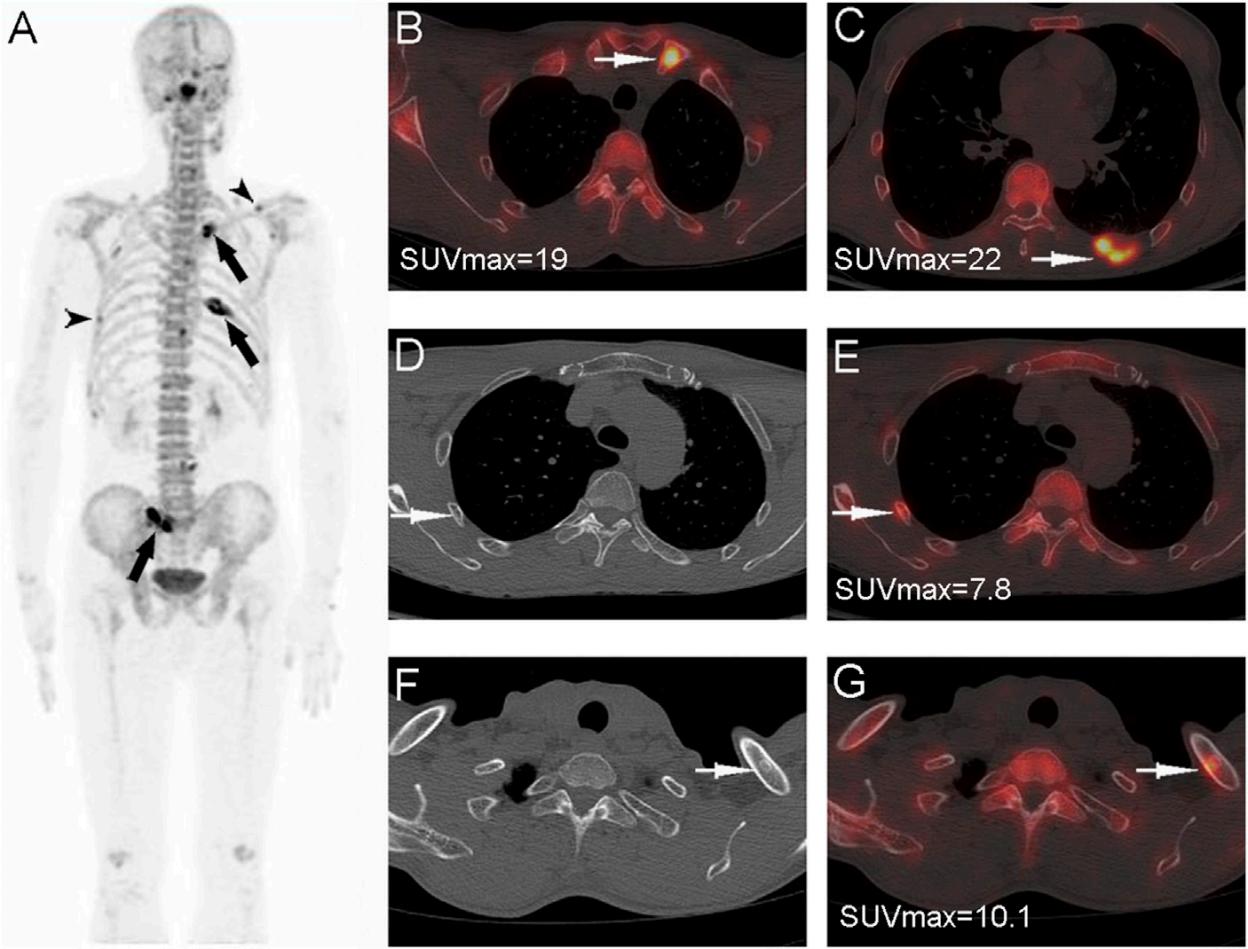
Nasopharyngeal carcinoma (T3N2M1) in a 55-year-old male patient. Whole-body PET MIP **(A)** shows multiple abnormally ^18^F-NaF uptake in multiple ribs, the right sacroiliac joint, and the left clavicle; the SUVmax of the confirmed bone metastases in the left clavicle **(B)**, PET/CT fusion, arrows and the left 8th posterior rib **(C)**, PET/CT fusion, arrows is significantly higher than that in the right rib **(D,E)**, arrows and the left clavicle **(F,G)**, arrows for benign lesions (non-fracture, non-osteophytes).

**FIGURE 6:**
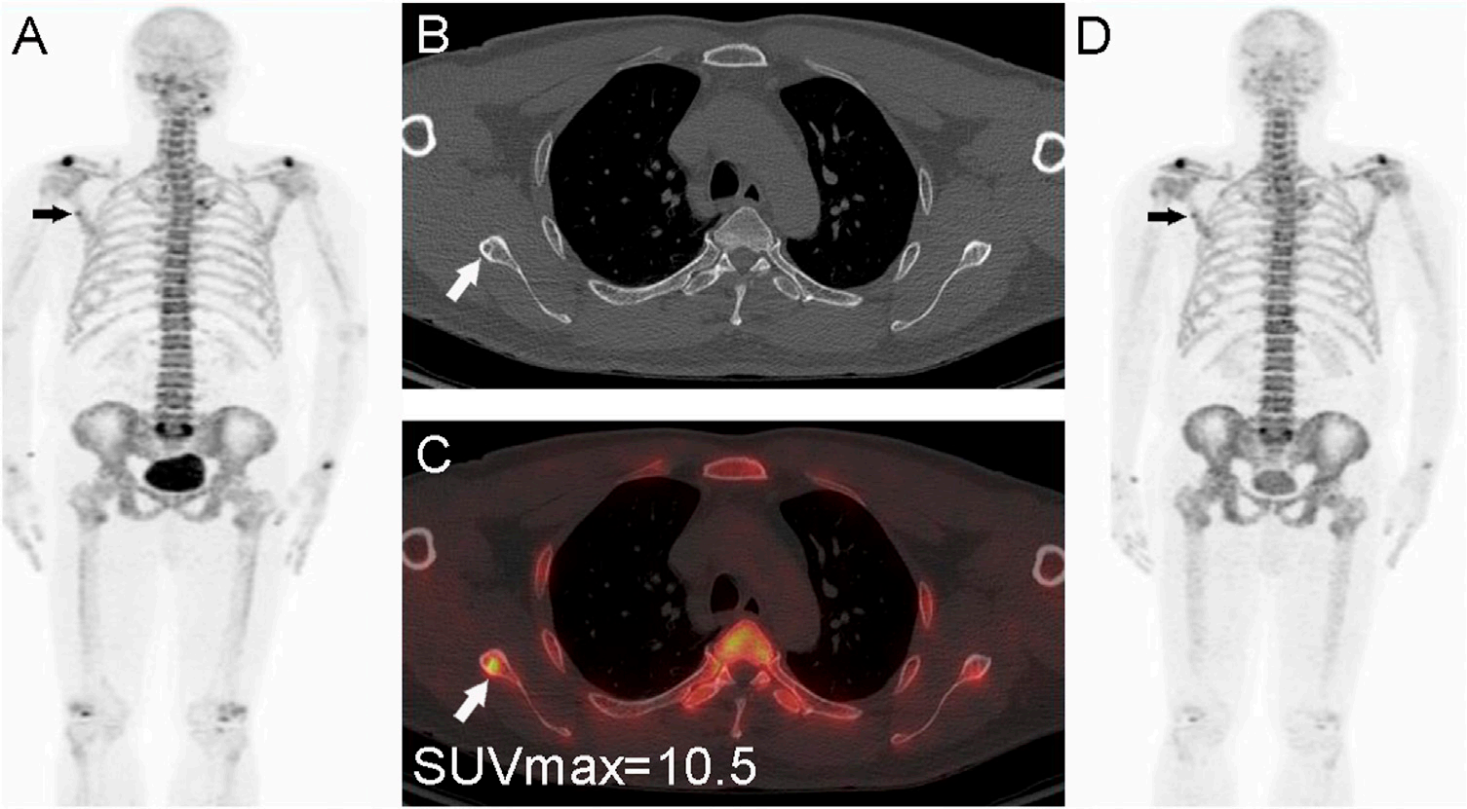
Nasopharyngeal carcinoma (T2N2M0) in a 56-year-old male patient. The whole-body PET MIP image **(A)**, arrow shows a focal mild abnormal ^18^F-NaF uptake in the right scapula, CT **(B)**, small arrow shows a small sclerotic border, PET/CT image **(C)** shows a localized ^18^F-NaF uptake, and measurement of the lesion with SUVmax = 10.5 < 12.5. The lesion was first considered benign based on the CT findings and the degree of metabolism. A follow-up PET/CT **(D)**, arrow at 1 year showed no metabolic changes and thus confirming the benign nature.

Quantitative analysis has the advantage of higher objectivity and reproducibility than visual qualitative analysis, and it has been widely used in the F-fluorodeoxyglucose (^18^F-FDG) PET/CT (Boellaard, 2009). Compared with the ^18^F-FDG PET/CT, ^18^F-NaF PET/CT is more advantageous for diagnosing bone metastases due to its higher sensitivity and specificity (Iagaru et al., 2013; Zhang et al., 2018). It has been suggested that quantitative analysis of ^18^F-NaF PET/CT findings could indicate a small coefficient of variation and good reproducibility, which could be used as a clinical reference for patient management (Sabbah et al., 2015; Lin et al., 2016). In addition, some scholars have quantitatively analyzed ^18^F-NaF PET/CT findings (SUVmax, SUVpeak) for the diagnosis of prostate cancer and other metastatic bone tumors, and evaluation of its efficacy after treatment revealed its potential application (Sabbah et al., 2015; Harmon et al., 2017; Muzi et al., 2021). Zhang et al. (Zhang et al., 2021) performed a quantitative single-photon emission computed tomography (SPECT)/CT-based study on 51 patients with malignant tumors, including 48 bone metastases and 40 benign bone lesions, and the results showed that the SUVmax of bone metastases (24.8 ± 16.3 g/ml) was significantly higher than that of benign lesions (15.9 ± 8.5 g/ml). The quantitative analysis of ^18^F-NaF PET/CT and ^99m^Tc-HDP SPECT/CT findings showed some similarities. In a study on the quantitative analysis of ^18^F-NaF PET/CT and ^99m^Tc-HDP SPECT/CT findings for detection of bone lesions at the same site in patients with prostate cancer and breast cancer, the SUVmax, SUVmean, SUVpeak, and SUV ratio of lesions to adjacent normal bone tissue were compared, and a strong positive correlation was found among the SUVs (SUVmax, SUVpeak, and SUVmean) (*R* ^2^ ≥ 0.80) (Arvola et al., 2019). The results of the present study showed that the SUVmax of malignant bone lesions (22.2 ± 9.4) was significantly higher than that of benign bone lesions (17.5 ± 14.9), especially non-fractured and non-osteophytes lesions (11.7 ± 5.4) (Figure 5). In addition, we found a higher SUVmax in benign bone lesions with osteophytes and fractures, which was not significantly different from the bone metastasis group (*p* = 0.268). In clinical practice, CT images from the PET/CT machine can well identify typical benign bone lesions, such as fractures, and osteophytes lesions, while there is a diagnostic challenge in the identification of malignant bone lesions and benign bone lesions, which are non-osteophytes and non-fractures, accompanied by the increased ^18^F-NaF uptake. Therefore, the advantage of SUVmax in distinguishing metastatic bone lesions from benign bone lesions (non-osteophytes, non-fractures) includes an effective complement to the discrimination of CT images. According to the ROC curves for groups 1 and 2.1, found that the diagnostic sensitivity (91.2%) was higher with SUVmax ≥12.5 as the threshold, and this result may provide a reliable reference for clinical practice (Figure 6).

The difference in SUVmax between different sizes of bone metastases in NPC based on ^18^F-NaF PET/CT has not been previously reported. The present study showed statistically significant differences in SUVmax between metastatic bone lesions of different sizes. The SUVmax of lesions in the diameter ≥2 cm group was significantly higher than that in the diameter <2 cm group, suggesting that SUVmax may be influenced by the size of lesions. The possible mechanism is that larger lesions contain more tumor cells, which release more relevant cytokines resulting in faster bone blood flow and bone ion exchange, thereby requiring more imaging agents, accompanied by a larger SUVmax; on the other hand, small lesions are affected by partial volume effects, which may also lead to underestimation of SUVmax. In addition, the difference in SUVmax between different patterns of bone metastases in NPC based on ^18^F-NaF PET/CT has also not been previously reported. We found that osteogenic bone metastases had significantly higher SUVmax values than osteolytic metastases. The main principle is that the bone structure of osteogenic bone metastases has more and denser calcium deposits than the original bone structure, the blood exchange of bone salts is accelerated, and more ^18^F-NaF imaging agent is taken up; whereas, in osteolytic bone metastases, the bone structure is destroyed, the calcium salts are reduced, the bone density is decreased, and the blood exchange of bone salts is slowed, so the NaF uptake is reduced.

In summary, after excluding osteophytes and fracture lesions based on CT findings, quantitative analysis of ^18^F-NaF PET/CT (SUVmax) can assist nuclear medicine physicians in the discrimination of metastatic bone lesions from benign bone lesions. Using SUVmax≥12.5 as the threshold can be a valuable reference for identifying bone metastases in NPC. The size of a metastatic bone lesion may affect SUVmax. This study has limitations. Although it is a PET/CT study of NPC in a large sample, however, it is a retrospective study and bias exists. Second, a histological sampling of all detected metastases is usually not required for determining the oncologic treatment concept (Bruckmann et al., 2021). Therefore, the reference standard was also based on follow-up examinations using CT, MRI and PET/CT.

## Conclusion

Visual analysis of ^18^F-NaF PET/CT findings is accompanied by high sensitivity and specificity for the diagnosis of bone metastases in NPC. Quantitative analysis of PET/CT (SUVmax) is valuable for the differential diagnosis of bone metastases in NPC. After excluding osteophytes and fracture lesions based on CT findings, using SUVmax ≥12.5 as the threshold can be an important reference for the differential diagnosis of bone metastases and benign bone lesions in patients with NPC. The next a multicenter prospective study is needed.

## Data Availability

The raw data supporting the conclusion of this article will be made available by the authors, without undue reservation.
